# It is Time to Renew!

**DOI:** 10.5935/1678-9741.20160046

**Published:** 2016

**Authors:** Domingo M. Braile

**Affiliations:** 1 Editor-in-Chief - BJCVS

Dear friends,

It is with great satisfaction that we deliver one more issue of the Brazilian Journal of Cardiovascular Surgery (BJCVS).

This has been a special year for all of us of the Editorial Board, besides celebrating our 30^th^ anniversary we are deeply restructuring the BJCVS.

In July 2015, during a meeting of the Board of the *Sociedade Brasileira de Cirurgia Cardiovascu* lar (SBCCV), we decided that the administrative system of the BJCVS should be directly managed by the SBCCV, which allowed a deep and systemic view of the journal and, measures could be taken, to reorganize the edition system and the administrative process.

After a year under this new management system, we can say that things are being organized. The change in nomenclature was formalized, the BJCVS can now be found on the Thomson Reuters website by its name in English: Brazilian Journal of Cardiovascular Surgery. All of you can access the website and enjoy the performance of your article in this database, from which the impact factor (IF) is calculated.

You just need to enter the link: http://goo.gl/VBhrgi, click "Sign in Web of Sciences", fill out the form or do log in, with your name and password.

On the next page, go to "Basic Search", type the journal name in English and select the index: Publication Name and "SEARCH". The articles appear in chronological order from the newest to the oldest, but there is a floating box that allows several possibilities.

Browsing the page, you will find a lot of important information, such as number of citations and viewing statistics.

As we want that BJCVS become known in many different strata, we updated our presence in all indexing bases. All failures in the editorial processes were identified and remedied by implementing a work schedule involving the chief editor, associate editors, reviewers, authors and the entire team of the BJCVS and SBCCV, with the responsibility of strict adherence to deadlines.

A checklist to optimize productivity was prepared, ensuring the reduction of time between manuscript submission and article publication.

Aimed at internationalization, we are working to bring the BJCVS to international standards and, from July, the authors must submit their manuscripts through the "ScholarOne" system, a platform used by the major journals in the world, a tool with a simple interface that will make it easier for authors and to editorial board, as well as allowing speeding up the entire review process. The BJCVS has now a blog with interviews, publication of interesting articles and works related to cardiovascular surgery together with tips for publications.

Despite many positive changes, there are still challenges to be overcome, such as to incentive the graduation and post-graduation institutions, encourage the authors to publish their scientific productions in the BJCVS, steadying our scientific and educational nature, among other important actions. We are convinced that all efforts made until now and those still to come, will bring good medium-term results, as the increased visibility of the BJCVS, with increased number of readers and citations, and consequently, increase of the "Thomson Reuters", IF. We would like to give special thanks the Board of the SBCCV that, at all times, are partners in our great fight, and in particular, the Editorial Board of the BJCVS, the authors, volunteers and reviewers, who work with primacy to give for the readers what is best in scientific production of our specialty.

We are proudly working to pave the way for the next 30 years of the BJCVS; after all, we are still the only Scientific Journal on Cardiovascular Surgery, with IF in the Southern Hemisphere!

We expect that the same enthusiasm permeates all our partners, because the most visible part of the work of each of us focuses on the BJCVS, our visiting card around the world!

Kind regards

**Domingo M. Braile** ^1^Editor-in-Chief - BJCVS

